# 4-phenyl butyric acid improves hepatic ischemia/reperfusion and affects gene expression of ABC transporter *Abcc5* in rats

**DOI:** 10.3325/cmj.2023.64.391

**Published:** 2023-12

**Authors:** Bülent Barış Güven, Alpaslan Tanoglu, Fatih Ozcelik, Esra Guzel Tanoglu, Neslihan Kaya Terzi

**Affiliations:** 1 Department of Anesthesiology, Dr. Suat Gunsel University of Kyrenia Hospital, Kyrenia, Cyprus; 2 Gastroenterology Division, Department of Internal Medicine, Bahcesehir University Faculty of Medicine, Istanbul, Turkey; 3 Department of Medical Biochemistry, University of Health Sciences Turkey, Hamidiye Etfal Training Hospital, Istanbul, Turkey; 4 Department of Molecular Biology and Genetics, University of Health Sciences, Istanbul, Turkey; 5 Department of Pathology, Canakkale Onsekiz Mart University, Faculty of Medicine, Canakkale, Turkey

## Abstract

**Methods:**

Thirty-five rats were randomly divided into five groups: sham, IR, IR + 100 mg kg^−1^ PBA, IR + 200 mg kg^−1^ PBA, and IR + placebo. After sacrifice, we assessed serum biochemical variables, myeloperoxidase (MPO), malondialdehyde (MDA), total antioxidant status (TAS), and total oxidant status (TOS). The expression levels of *Abcc (2 and 5), Abcg2*, *Abcf2, Ire1-α, and Perk* genes were measured with a quantitative real-time polymerase chain reaction.

**Results:**

Serum biochemical variables, MPO, MDA, TAS, and TOS levels of the PBA groups (especially in the low dose group) were lower than in the IR and placebo group (*P* < 0.05). Histological tissue damage in the IR group was more severe than in the PBA groups. *Ire1-α* and *Perk* expression levels were significantly lower in the PBA groups than the IR group (*P* < 0.001). *Abcc* (2 and 5) and *Abcg2* expression levels were significantly lower in the IR group than in the sham and PBA groups (*P* < 0.001, *P* < 0.035, and *P* < 0.009, respectively).

**Conclusions:**

The use of PBA significantly positively affected IR injury, which makes PBA a candidate treatment to reduce liver IR.

Correspondence to: Bülent Barış Güven Girne Mah., Madenci sok. 16 Whitecity sit, D-14 Maltepe, 34852, Istanbul, Turkey *barguv@gmail.com*

An important role in ischemia reperfusion (IR) injury is played by systemic inflammation and the related release of endothelial factors, free oxygen radicals, and neutrophils (1-4). With the initiation of reperfusion, free oxygen radicals (FOR) are released from leukocytes, and endothelial and parenchymal cells. FOR impair the permeability of the mitochondria and cause cell death by inhibiting the cell's adenosine triphosphate (ATP) production. The mediators responsible for IR injury are FOR, lipid peroxidation, and inflammatory cells (5). In addition, during FOR formation there is an increase in tumor necrosis factor-alpha (TNF-α) (6-8).

IR injury is one of the important causes of graft dysfunction in liver and other solid organ transplantations (9-11). Therefore, it is essential to develop strategies to prevent or mitigate IR injury in transplantation cases. Liver ischemia may also occur in cases of trauma, cancer, bile duct obstructions, stricture operations, and following a hemodynamic or cardiogenic shock without surgical intervention (2,3). Many studies have assessed ways to reduce IR injury (12,13), but there is still no effective treatment.

Various pharmacological treatments are being investigated for their ability to increase the efficacy of liver transplants, especially in the case of marginal donors (14-16). Different therapeutic agents can be used to reduce oxidative stress caused by ischemia in order to mitigate IR injury in organ transplantation. Studies investigating these agents used different models of transient ischemia and syngeneic or allogeneic transplants (9,10,17).

IR injury damages the endoplasmic reticulum (ER), which plays a central role in lipid biosynthesis and protein folding. Such stress in the ER influences programmed cell death (18). One of the mediator chemicals that can prevent ER stress in different cell types is 4-phenyl butyric acid (PBA) (19). PBA was shown to suppress inflammatory processes and cell proliferation, reduce oxidative stress, and increase the expression of some important metabolic genes (18-20). It was approved by the FDA for use in humans at doses of 7-15 g/day in some hyperammonemia conditions such as urea cycle disorders. In addition, it has been used for the treatment of sickle cell disease, cystic fibrosis, and some neurodegenerative diseases and cancers (18,19).

ATP-binding cassette (ABC) transporter genes are a superfamily of integral membrane proteins. They are divided into seven different subtypes, from ABC A to G, according to the ATP-binding amino acid sequence (21). The ABC transporter gene family is expressed in nearly all tissues and cells as a marker of cellular defense against xenobiotics and their metabolites. These genes take part in cleaning metabolic waste and foreign compounds in the kidney and liver tissues (22). ABC transporters are active carrier proteins of many substances, such as amino acids, polysaccharides, lipids, chemicals, drugs, and toxins (23). In addition, they protect the cell from apoptosis and hypoxic injury (24,25). The effect of PBA on ABC transporters has been understudied. Therefore, this study aimed to examine the effect of PBA treatment on oxidative stress markers, inflammation, liver function tests, liver histology, ER stress, and expression levels of ABC gene family members in an experimental liver IR model.

## MATERIAL AND METHODS

### Experimental animals

The study was carried out in the Istanbul Experimental Animal Laboratory of the University of Health Sciences, Turkey in December 2021. The study involved adult (16-20 weeks old) male Sprague-Dawley rats weighing on average 250-300 g. The animals were housed in laboratory conditions at an ambient temperature of 20-22 °C, under 12:12 light cycle. The study was approved by the Animal Experiments Local Ethics Committee of the University of Health Sciences, Turkey (2018-05/17), and all the experimental procedures complied with the Helsinki Declaration.

Thirty-five rats were randomly distributed to five study groups by using a computer-based randomization program, as follows:

Sham group (n = 7): Only laparotomy was performed under general anesthesia; no IR was induced.

IR control group (n = 7): IR was induced and no other intervention was performed.

IR+4-PBA 100 mg kg^−1^ (n = 7): 100 mg kg^−1^ PBA (Sigma-Aldrich, St. Louis, MO, USA) was administered intraperitoneally 1 hour before anesthesia, and the IR protocol was applied.

IR+4-PBA 200 mg kg^−1^ (n = 7): 200 mg kg^−1^ PBA was administered intraperitoneally 1 hour before anesthesia, and the IR protocol was applied.

Placebo (n = 7): 1 mL of saline was administered intraperitoneally 1 hour before anesthesia, followed by IR protocol, and no other medication was administered (Table 1).

**Table 1:** Study groups and protocol implementation

	Ischemia-Reperfusion (IR)	4-phenyl butyric acid (PBA)	Saline	Laparotomy
Sham group, n = 7	-	-	-	+
IR group, n = 7	+	-	-	+
**PBA** 100 mg/kg, n = 7	+	100 mg/kg	-	+
**PBA** 200 mg/kg, n = 7	+	200 mg/kg	-	+
**Placebo**, n = 7	-	-	1 mL	+

Laparotomy was performed in the following way. The anterior abdominal wall of the rats was cleaned by gently shaving, and a midline incision was made to approach the liver. To remove the intestines after laparotomy in the IR, PBA, and placebo groups, the cecum was pulled toward the left forefoot. The portal vein and hepatic artery located under the liver were gently clamped without injury by using a microvascular clamp. The procedure lasted 120 minutes. In liver IR models in general, partial (20%, 50%), subtotal (70%), or total (100%) hepatic ischemia are induced, and the acceptable warm ischemia duration is 45-60 minutes (26,27). In our study, we used a total ischemia model, and the duration of ischemia was 60 minutes. The duration of reperfusion was also 60 minutes (26,27).

The rats were euthanized by drawing blood from the heart under general anesthesia. The protocol used for general anesthesia during surgical procedures and IR was the same in all groups. Intraperitoneal ketamine (100 mg kg^−1^) and xylazine (10 mg kg^−1^) were used. Since the experiment was long, 1/3 of the initial dose was administered at one-hour intervals as a maintenance dose.

The study was terminated after the liver was removed and the tissue sample was obtained. The total working time was the same for all groups. One hour after drug administration, the microvascular clamp was used to block the hepatic blood.

Some of the liver tissue samples taken after the experiment were placed into 10% formaldehyde solution. They were examined histopathologically in the pathology laboratory, and the Ki67 proliferation index was determined. The rest of the liver tissues were placed in microcentrifuge tubes with lock caps and underwent myeloperoxidase (TMPO) measurement in the biochemistry laboratory. The blood samples were centrifuged, and the obtained serum was kept at -80 °C until analysis.

Tissues taken for TMPO measurement were lysed with an ultrasonic homogenizer (Scientific Industries SI, Bohemia, NY, USA; Disruptor Genie, 2800 rpm and 15 min) in 2-mL Tris-buffer at +4 °C (homogenized as 50 mg with 500 μL PBS) and stored at -80 °C. Investigators who performed statistical, genetic, biochemical, and histopathological analyses were blinded to group assignment, except those who collected blood and tissue samples.

### Biochemical analysis

Serum aspartate aminotransferase (AST), alanine aminotransferase (ALT), albumin, malondialdehyde (MDA), myeloperoxidase (MPO), total antioxidant status (TAS), insulin-like growth factor 1 (IGF-1), total oxidant status (TOS), TNF-α, and liver TMPO were measured with a microplate ELISA device (BioTek Epoch-2 Spectrophotometer, Highland Park, IL, USA). Measurements were made according to the test procedure, and double wells were used for both standard and sample tests. Unbound streptavidin-HRP was removed after incubation and washing. Subsequently, a substrate solution containing TMP was added. After the color developed, an acid-stopping solution was added. Optical density was measured as absorbance at the 450-nm wavelength.

### Histopathological and immunohistochemical examinations

The liver tissue collected for histopathological examination was fixed in formalin and embedded in paraffin. From the prepared paraffin tissues, 3-μm thick sections were cut with a microtome. Hematoxylin-eosin-stained slides were scored according to Suzuki (28) (hepatic sinusoidal congestion degree, cytoplasmic vacuolization degree, and parenchymal cells necrosis degree). The paraffinized sections were prepared for immunohistochemical staining. After deparaffinization, the sections were rehydrated in graded ethanol solutions. Following antigen binding in a pressure cooker containing EDTA/Tris buffer (pH 9.0), endogenous peroxidase activity was blocked by exposure to 20% H_2_O_2_ for 15 minutes. A two-hour incubation was used for the primary antibody, Ki67 (Dako Corporation, Carpinteria, CA, USA). Ki67 expression in positively stained cells was determined by considering nuclear reactivities only in the microscopic field (using 40 × magnification) and identifying the areas of positive Ki67 (brown-colored cells). Ki-67 proliferation index values 0%-1% were considered as low proliferation, 2%-5% as medium proliferation, and 6%-7% as high proliferation.

### RNA isolation

Total RNA was isolated from equal volumes (500 mg) of tissue samples by using TRIZol (Thermo Fisher Scientific, Whaltam, MA USA). The purity and concentrations of RNA samples were determined with a spectrophotometric method by using Denovix DS-11 (Wilmington, DE, USA).

### Polymerase chain reaction and cDNA synthesis

One microgram of RNA was reverse-transcribed by using Transcriptor High Fidelity cDNA (Roche Diagnostics, Mannheim, Germany) synthesis kit as per the manufacturer's protocol. Quantitative real-time PCR (qRT-PCR) was performed on a LightCycler 480-II real-time thermal cycler (Roche, Basel, Switzerland) by using Roche's SYBR Green Master Mix. The primer sequences are shown in Table 2. The reactions were performed at 95 °C for 10 minutes, followed by 40 cycles at 95 °C for 15 seconds, and 40 cycles at 60 °C for 1 minute. The data for gene expressions were normalized with GAPDH (Abcam plc, Cambridge, UK).

**Table 2:** Primer sequences used for real-time polymerase chain reaction analysis

Gene name	Forward (5′-3′)	Reverse (5′-3′)
***Abcg2***	AGTCCGGAAAACAGCTGAGA	CCCATCACAACGTCATCTTG
***Abcc2***	CTGGTTGGAAACTTGGTCG	CAACTGCCACAATGTTGGT
***Abcc5***	AACAGGAAGGATTCTCAACAGG	TGAATGCTGGACGTGATATGG
***Abcf2***	GAGGTTTCACTGGGAGCAAGATC	CTGTAGCGTCTTCTCCTTGCTC
***Ire1-α***	CCTGAGGAATTACTGGCTTCTC	TCCAGCATCTTGGTGGATG
***Perk***	CGCTGCTGCTGCTGTTCCTG	GCAATGCCTCGGCGTCTTCC
***Gapdh***	TATCGGACGCCTGGTTAC	CTGTGCCGTTGAACTTGC

### Statistical analysis

Since the number of rat groups was more than two and the number of animals in the groups was fewer than 30, nonparametric statistical tests were used. A Kruskal-Wallis test was performed to assess the differences between the groups. A *post-hoc* Dunn's multiple comparisons test was used when there was a difference between the groups (*P* < 0.05). The differences between the groups in qRT-PCR results were assessed with a one-way ANOVA test. A correlation matrix analysis was used to evaluate the relationships between the data. Spearman correlation analysis was used for parametric data. The statistical analysis was performed with SPSS, version 16.0 (SPSS, Chicago, IL, USA) and InStat3 Statistics (San Diego, CA, USA).

## RESULTS

### Biochemical analysis

**Figure 1:**
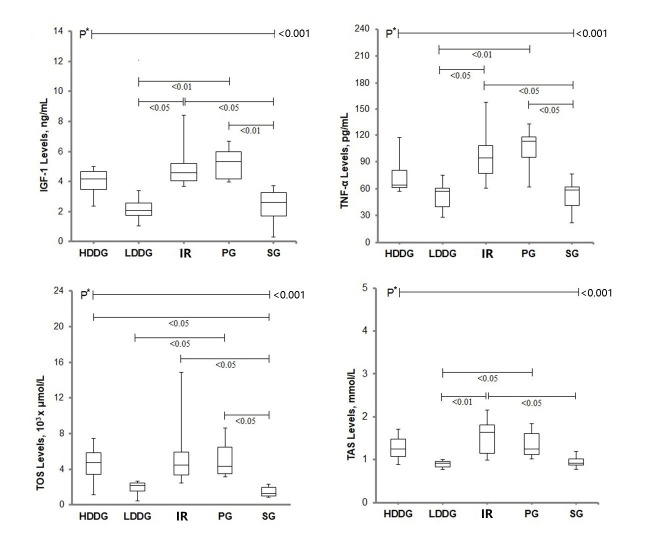
Comparison between the groups in terms of insulin-like growth factor 1 (IGF-1), tumor necrosis factor alpha (TNF-α), total antioxidant status (TAS), and total oxidant status (TOS) levels using Kruskal-Wallis test* with a *post-hoc* test. HDDG – high-dose drug group, LDDG – low-dose drug group, IR – ischemia- reperfusion group, PG – placebo group, SG – sham group.

*IGF-1 and TNF-α levels.* IGF-1 and TNF-α levels in the low-dose group (LDDG) were significantly lower than in the IR and placebo group (*P* < 0.05) and similar to those in the sham group (Table 3, Figure 1).

**Table 3:** Biochemical analysis results according to groups^†^

	4-phenyl butyric acid 200 mg/kg	4-phenyl butyric acid 100 mg/kg	Ischemia-reperfusion	Placebo	Sham	*P* Values
**N**	7	7	7	7	7	-
Insulin-like growth factor, ng/mL	4.17 (2.36-5.01)	2.09 (1.04-3.38)	4.59 (3.68-8.39)	5.34 (3.97-6.67)	2.61 (0.29-3.72)	0.0001*
Tumor necrosis factor alpha, pg/mL	63.9 (57.1-117.3)	56.9 (28.4-75.2)	94.0 (60.4-157.7)	112.7 (61.9-131.9)	58.1 (22.2-76.4)	0.0007*
Total antioxidant status, mmol/L	1.24 (0.89-1.71)	0.92 (0.77-1.00)	1.63 (0.99-2.15)	1.24 (1.01-1.84)	0.91 (0.78-1.19)	0.0007*
Total oxidant status, μmol/L	4716 (1125-7429)	2201.90 (422-2669)	4502 (2469-14894)	4351 (3144-8645)	1310 (891-2310)	0.0002*
Total bilirubin, mg/dL	0.90 (0.31-1.53)	0.38 (0.19-0.54)	1.20 (0.38-2.05)	0.94 (0.62-1.55)	0.39 (0.03-0.85)	0.0013*
Direct bilirubin, mg/dL	0.14 (0.02-0.40)	0.05 (0.01-0.24)	0.22 (0.02-0.51)	0.14 (0.05-0.37)	0.07 (0.01-0.22)	0.1516*
Albumin, g/L	22.6 (11.3-26.5)	22.7 (19.6-24.7)	23.3 (13.6-31.3)	24.93 (12.3-26.4)	23.3 (20.0-24.7)	0.5092*
Alanine aminotransferase, IU/L	151 (88-202)	107 (92-189)	302 (211-377)	279 (185-383)	56 (34-71)	0.0062*
Aspartate aminotransferase, IU/L	133 (96-186)	114 (90-150)	316 (183-369)	342 (202-401)	55 (38-66)	0.0028*
Serum myeloperoxidase, ng/mL	19.8 (17.4-27.9)	20 (16.9-25.2)	40.3 (29.9-48.8)	36.9 (30.5-45.1)	13.2 (9.2-15.9)	0.0010*
Malondialdehyde, ng/mL	42.3 (37.2-55.1)	40.5 (37.1-50.6)	83.3 (68.7-98.1)	87.8 (70.6-99.9)	30.3 (20.6-35.5)	0.0064*
Liver tissue myeloperoxidase, ng/mL	100.3 (82.5-129.2)	90.1 (74.4-116.8)	150.9 (119.4-172.2)	161.3 (130.6-189.1)	50.1 (40.5-65.8)	0.0015*

*Kruskal-Wallis test (nonparametric ANOVA) with a *post-hoc* Dunn’s multiple comparisons test.

†Results are expressed as the median (min-max).

*TAS and TOS levels.* TOS levels in the LDDG were significantly lower than in the IR group (*P* < 0.05) and the placebo group. TAS levels were also significantly lower in the LDDG than in the IR group and placebo group (*P* < 0.01 and *P* < 0.05, respectively) (Table 3, Figure 1). Low-dose PBA, unlike high-dose PBA, significantly affected TAS, and TAS levels were decreased to the level similar to the sham group. This finding corresponds with the TOS data because we observed a very low oxidative state in the LDDG. In addition, this finding is supported by the positive correlation between TAS and TOS (Table 4).

**Table 4:** Correlation matrix results for the variables that were significantly different between the groups

n = 35	Insulin-like growth factor 1	Tumor necrosis factor alpha	Total antioxidant status	Total oxidant status	Alanine aminotransferase	Total bilirubin	Ki-67
Insulin-like growth factor 1	1.0000						
Tumor necrosis factor alpha	0.4547	1.0000					
Total antioxidant status	0.5966	0.3879	1.0000				
Total oxidant status	0.6211	0.3544	0.7972	1.0000			
Alanine aminotransferase	0.4485	0.4043	0.4196	0.3019*	1.0000		
Total bilirubin	0.5955	0.4457	0.7419	0.6878	0.2588*	1.0000	
Ki-67	0.4353	0.4594	0.5477	0.3653	0.4365	0.4680	1.000

*P > 0.05, other correlations *P* < 0.05. Since the correlation matrix results showed a Gaussian distribution, Pearson r correlation analysis was used. Correlation coefficients of 0.01-0.29 were interpreted as a low-level relationship, 0.30-0.70 as a moderate relationship, 0.71-0.99 as a high-level relationship, and 1.00 as a perfect relationship.

**Figure 2:**
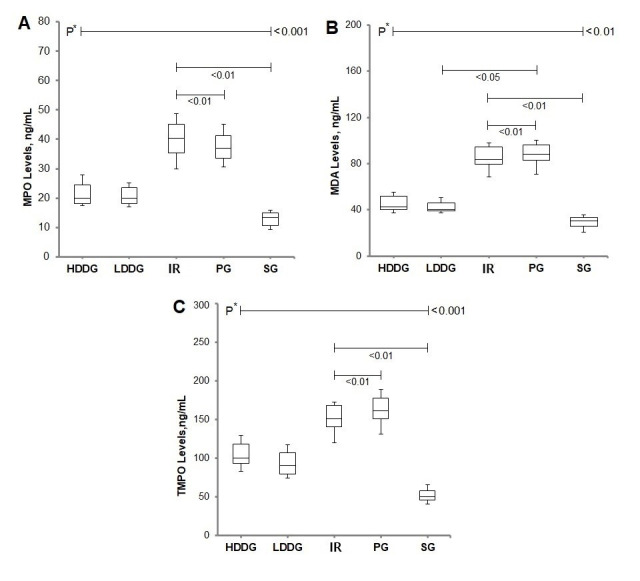
Comparison of groups in terms of serum myeloperoxidase (MPO), malondialdehyde (MDA), and liver tissue myeloperoxidase (TMPO) levels with a Kruskal-Wallis test* with a *post-hoc* test. HD – high-dose drug group, LD – low-dose drug group, IR – ischemia- reperfusion group, P – placebo group, S – sham group.

*MPO, TMPO, and MDA levels.* MPO levels were significantly lower in the LDDG compared with the IR group (*P* < 0.01). Moreover, differences between the placebo group and treatment groups were also significant. TMPO levels were significantly lower in the treatment groups than in the IR group (*P* < 0.05). These results are compatible with serum MPO levels. When it comes to MDA levels, they were also significantly lower in the treatment groups than in the IR and placebo group (both *P* < 0.01) (Figure 2).

**Figure 3:**
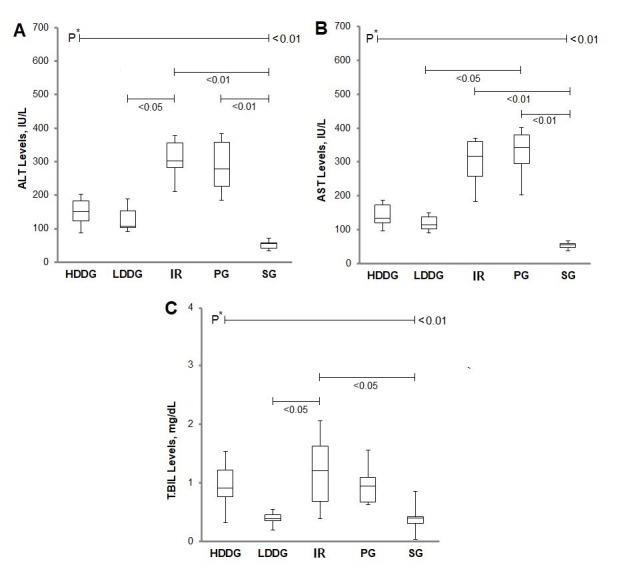
Comparison of groups in terms of aspartate aminotransferase (AST), alanine aminotransferase (ALT), and total bilirubin (T.BIL) levels with a Kruskal-Wallis test* with a *post-hoc* test. HD – high-dose drug group, LD – low-dose drug group, IR – ischemia- reperfusion group, P – placebo group, S – sham group.

*AST, ALT, albumin, and bilirubin levels.* ALT and AST levels were significantly lower in the treatment groups than in the IR and placebo group (*P* < 0.01) (Table 3, Figure 3). Total bilirubin levels significantly increased in the IR and placebo groups; however, they significantly decreased after PBA treatment (*P* < 0.01). Moreover, total bilirubin levels in the LDDG were similar to those in the sham group. Finally, there was no difference in albumin and direct bilirubin levels. Significant positive correlations were found between IGF-1, TNF-α, TAS, TOS, ALT, and Ki-67 (Table 4).

### Histopathological examination results

**Figure 4:**
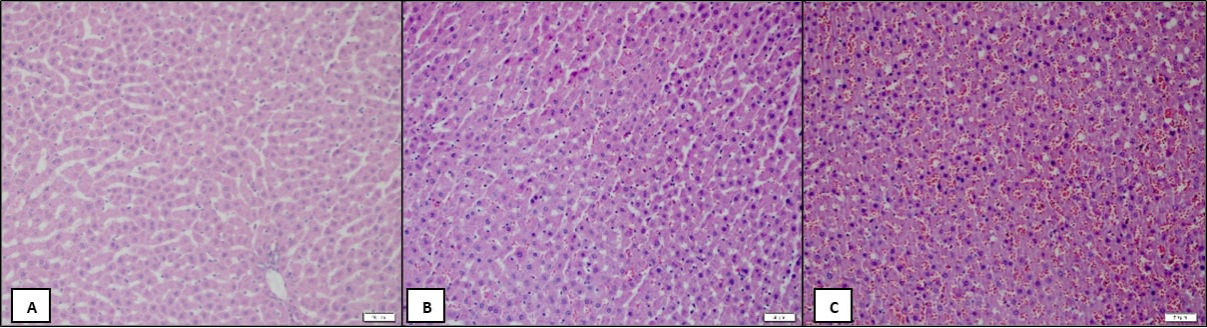
Hematoxylin-eosin (HE) staining of liver tissue samples. (**A**) Sham group (cytoplasmic vacuolization and parenchymal necrosis are not visible) (HE, ×40) (**B**) Low-dose 4-phenyl butyric acid group (minimal sinusoidal congestion, minimal single-cell necrosis and minimal neutrophil infiltration are present) (HE, ×40) (**C**) Ischemia-reperfusion group (necrosis and severe sinusoidal congestion are present). Neutrophil infiltration in parenchymal areas, moderate sinusoidal congestion, moderate cytoplasmic vacuolization, and pale-stained, damaged hepatocytes specific to ischemic necrosis are visible (HE, ×40).

Hepatocyte vacuolization degree and hepatic sinusoidal congestion degree were significantly lower in the treatment groups than in the IR group (*P* < 0.05) and the placebo group (Table 5). Hematoxylin-eosin staining of liver tissue samples is shown in Figure 4.

**Table 5:** Histopathological findings in different study groups^†^

	High-dose 4-phenyl butyric acid group	Low-dose 4-phenyl butyric acid group (LDDG)	Ischemia- reperfusion (IR)	Placebo (PG)	Sham group (SG)	*P* Values*
N	7	7	7	7	7	-
Degree of hepatic sinusoidal congestion, n	2 (1-2)	2 (0-2)	4 (3-4)	4 (3-4)	1 (0-1)	0.019
Comparison	LDDG-IR: *P* < 0.05, LDDG-PG: *P* < 0.05, IR-SG: *P* < 0.05, others: *P* > 0.05					
Degree of hepatocyte vacuolation, n	2 (1-3)	1 (1-2)	4 (3-4)	3 (2-4)	0 (0-0)	0.012
Comparison	LDDG-IR: *P* < 0.05, IR-SG: *P* < 0.05, PG-SG: *P* < 0.05, others: *P* > 0.05					
Degree of hepatocyte necrosis, n	1 (1-2)	1 (1-2)	3 (2-4)	3 (2-4)	0 (0-0)	0.013
Comparison	IR-SG: *P* < 0.05, PG-SG: *P* < 0.05, others >0.05					
Ki-67, %	3 (2-4)	2 (1-3)	5 (4-7)	4 (4-7)	1 (0-3)	0.0004
Comparison	LDDG-IR: *P* < 0.01, LDDG-PG: *P* < 0.05, IR-SG: *P* < 0.01, PG-SG: *P* < 0.01, others: *P* > 0.05					

*Kruskal-Wallis Test (nonparametric ANOVA) with post-hoc Dunn’s multiple comparisons test. The p value is approximate (from χ^2^ distribution). †Results are expressed as the median (min-max).

### Ki-67 index

The Ki-67 index was lower in the LDDG and the sham group than in the IR group (*P* < 0.01) (Table 5). Accordingly, it may be considered that PBA has a positive effect on cell proliferation.

### ABC transporter gene expression levels

**Figure 5:**
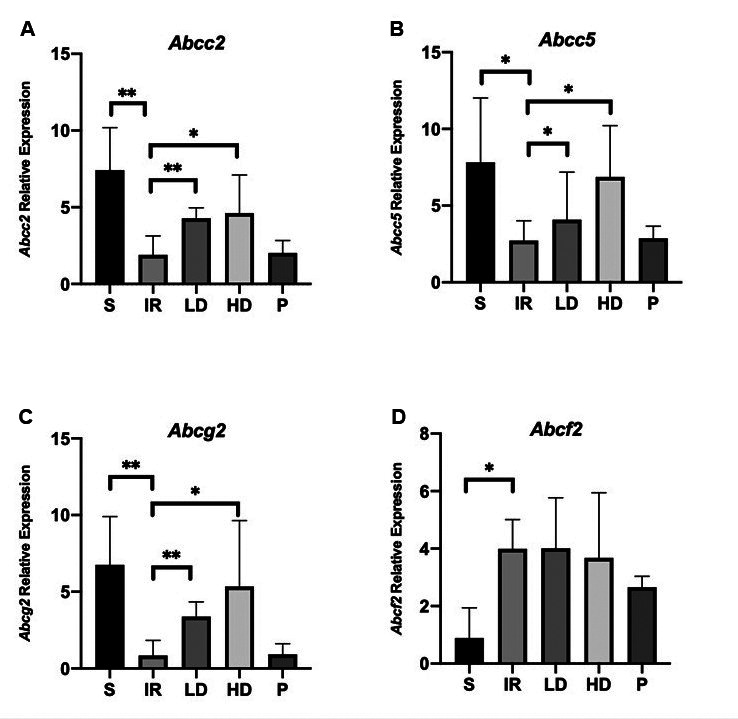
The expression levels of *Abcc2* (**A**), *Abcc5* (**B**), *Abcg2* (**C**), and *Abcf2* (**D**) genes in the study groups. * *P* < 0.05, ** *P* < 0.01, *** *P* < 0.001; Kruskal-Wallis test with a *post-hoc* test. HDDG – high-dose drug group, LDDG – low-dose drug group, IR – ischemia- reperfusion group, PG – placebo group, SG – sham group.

The expression of *Abcc2, Abcc5,* and *Abcg2* genes was significantly lower in the IR group than in the sham group (Figure 5A, Figure 5B, Figure 5C). In the LDDG, the levels of these genes significantly increased compared with the IR group and the placebo group (*P* < 0.05 for all genes) (Figure 5A, Figure 5B, Figure 5C). The expression of the *Abcf2* gene was higher in the IR group than in the sham group, but there was no significant difference between the IR group and the treatment groups (Figure 5D).

**Figure 6:**
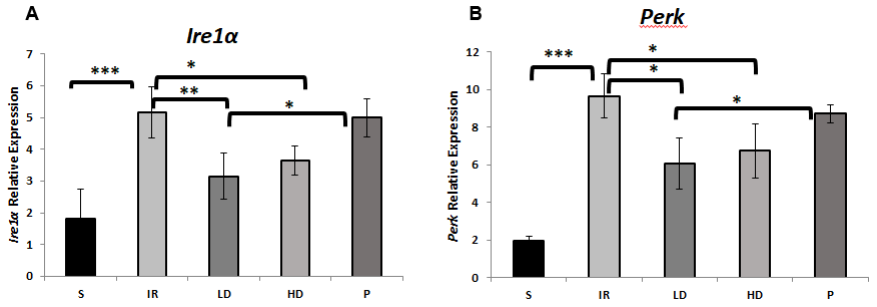
*Ire1-α* (**A**) and *Perk* (**B**) expression levels in the study groups. *P < 0.05, **P < 0.01, ***P < 0.001; Kruskal-Wallis test with a *post-hoc* test. HDDG – high-dose drug group, LDDG – low-dose drug group, IR – ischemia- reperfusion group, PG – placebo group, SG – sham group.

*IRE1-α and Perk gene levels. Ire1-α* was significantly higher in the IR group than in the sham group (*P* < 0.001). However, in the low-dose and high-dose treatment group, *Ire1-α* expression significantly increased compared with the IR group (*P* < 0.01 and *P* < 0.05, respectively) and the placebo group (*P* < 0.05) (Figure 6A). *Perk* gene levels were also significantly increased in the IR group compared with the sham group (*P* < 0.001). However, in the low-dose and high-dose treatment group, *Perk* expression significantly increased compared with the IR group (*P* < 0.05 and *P* < 0.05 respectively) and the placebo group (Figure 6B).

## DISCUSSION

In this study, we found significantly lower serum ALT, AST, IGF-1, TNF-α, MPO, MDA, TAS, and TOS levels in the treatment groups compared with the IR and placebo groups. The degree of vacuolization and sinusoidal congestion and TMPO levels; expression levels of *Abcc* (2,5), *Abcg2*, *Ire1-α*, and *Perk* genes; and Ki-67 proliferation index were also lower. All these results support the positive effects of PBA on the liver IR modeling.

To date, different experimental IR models have been used to assess the effects of various hepatocyte-protective agents (allopurinol, α-tocopherol, glucagon, melatonin, carnitine, aprotonin, catalase, aspartic acid, ubiquinone, etc) that block the pathophysiological mechanisms responsible for IR damage (29). The therapeutic agent used in our study was PBA. PBA is a pharmacological chaperone that has consistently demonstrated the ability to rescue the expression of folding and trafficking mutations in ABC transporters (22,23). In addition, since PBA has been clinically used for years in the treatment of some diseases, we also had sufficient knowledge about its pharmacodynamics. The PBA doses used in previous studies ranged from 20-300 mg kg^−1^ (20,30). Thus, in the current study, we used two different doses of PBA (100 and 200 mg kg^−1^).

We found significantly lower levels of IGF-1 and TNF-α, indicators of systemic inflammation, in the LDDG compared with the IR and placebo groups. IGF-1 and TNF-α levels in the LDDG were similar to those in the sham group, a finding that indicates the ability of PBA to suppress inflammation. TAS levels in the LDDG group were as low as in the sham group. This finding is consistent with the TOS results. We observed a very low oxidative status in rats treated with low-dose PBA, as well as a low antioxidative status. This is an expected finding because a low antioxidative status compensates for a low oxidative status. Contrary to this, when the oxidative load is high, the organism is expected to respond by increasing its antioxidant capacity. The positive correlation between TAS and TOS in our study supports this finding and shows the positive effects of our therapeutic agent on the IR status. In addition, we found significantly lower levels of MPO and TMPO in the PBA groups compared with the IR group. All these results show that PBA exhibits dose-dependent anti-inflammatory effects and reduces oxidative stress in liver IR injury. Vilatoba et al (20) also showed that PBA reduced inflammation and apoptosis in liver IR injury in a dose-dependent manner.

In our study, PBA showed beneficial histopathological effects on hepatocyte vacuolation and hepatic sinusoidal congestion degree. It also showed a positive effect on Ki-67 proliferation index, a good indicator of proliferation, which was higher in the IR group than in the low-dose PBA group. Significant positive correlations were found between the Ki-67 results and TAS, TOS, and inflammatory markers. Similar to our results, some recent studies have found that reactive oxygen species and reactive nitrogen species played an important role as signal molecules in programmed cell death, regulation of antioxidant responses, and cell proliferation (31,32).

Liver ER stress in various pathological circumstances is associated with ischemia, proinflammatory cytokines, the use of alcohol and other toxic compounds, metabolic disequilibrium, hepatotropic virus infections, and so forth (33,34). ER stress related to unfolded or misfolded proteins poses a fundamental threat to living liver cells. Several studies have shown that PBA decreased IR injury by reducing ER stress (20,35,36). In the current model of total liver IR, the expression of *Ire1-α* and *Perk* genes, indicating ER stress, was significantly decreased in both treatment groups. In other words, we showed that PBA alleviated liver IR injury by decreasing ER stress.

ABC transporter genes are ATP-dependent membrane proteins mostly expressed in the liver, gut, blood-brain barrier, prostate, and kidney (37,38). In the current study, we selected four ABC transporter genes (*Abcc2, Abcc5, Abcf2,* and *Abcg2*) based on a literature search. All these ABC transporter gene are expressed in the liver and in many processes in the liver such as inflammatory response and oxidative stress (39,40). In a previous IR study on the mouse liver, *Abcg2* and *Abcc2* gene levels were decreased seven days after liver ischemia (22). In another study, the expression of *Abcc2* in the liver was controlled by nuclear receptor activation, which is impaired during cholestasis (41). Abcg2 was found to play a role during oxidative stress, and its expression levels changed after IR injury in the kidney, liver, heart, cerebral vascular tissue, and intestines (42-47). The levels of *Abcc2* (*Mrp2*) expression in the rat liver decreased after IR injury, and endocytosis of *Mrp2* developing in the canalicular membrane following IR caused impaired bile function (48). Another study showed that *Abcc2* mRNA expression in the rat liver tissue was higher in the IR injury group compared with the sham group under the conditions of four-hour reperfusion after ischemia (49). Another study suggested that the changes in *Mrp2* expression levels may be an important determinant of cholestasis in ischemic livers (50). In the current study, *Abcc2* and *Abcg2* expression levels were significantly suppressed in the IR group compared with the sham group but significantly increased in the PBA treatment groups compared with the IR group. These findings indicate a beneficial effect of PBA in liver IR.

An important ABC transporter gene located in the liver is *Abcc5* (*Mrp5*) (51). No study so far has evaluated *Abcc5* expression levels in liver IR injury. According to a review article by Borst et al, the physiological functions of *Abcc5* remain to be investigated (52). For the first time in the literature, we found that *Abcc5* expression levels were significantly suppressed in the liver IR group compared with the sham group, but significantly increased in the PBA treatment groups compared with the IR group.

There are some limitations to our study. First, our study is a preliminary analysis, and its results need to be confirmed by further detailed studies. Moreover, this investigation was performed in a single experimental model, and extrapolation from rats to humans is difficult.

In conclusion, liver IR injury causes serious adverse effects through oxidative and ER stress. The use of PBA in IR injury affected the levels of the ABC transporter genes (*Abcc2*, *Abcc5*, and *Abcg2*), significantly suppressed inflammation and oxidative stress, reduced ER stress, and thus significantly mitigated liver IR injury. PBA can be recommended for use at an appropriate non-toxic dose to reduce liver IR.

## References

[1] Kim YI Ischemia-reperfusion injury of the human liver during hepatic resection. J Hepatobiliary Pancreat Surg 2003 10 195 9 10.1007/s00534-002-0730-x 14605975

[2] Konishi T Lentsch AB Hepatic ischemia/reperfusion: mechanisms of tissue injury, repair, and regeneration. Gene Expr 2017 17 277 87 10.3727/105221617X15042750874156 28893351 PMC5885149

[3] Kupiec-Weglinski JW Busuttil RW Ischemia and reperfusion injury in liver transplantation. Transplant Proc 2005 37 1653 6 10.1016/j.transproceed.2005.03.134 15919422

[4] Nakazato PCG Victorino JP Fina CF Mendes KDS Gomes MCJ Evora PRB Liver ischemia and reperfusion injury. Pathophysiology and new horizons in preconditioning and therapy. Acta Cir Bras 2018 33 723 35 10.1590/s0102-865020180080000008 30208134

[5] Deng WS Xu Q Liu YE Jiang CH Zhou H Gu L Effects of melatonin on liver function and lipid peroxidation in a rat model of hepatic ischemia/reperfusion injury. Exp Ther Med 2016 11 1955 60 10.3892/etm.2016.3160 27168834 PMC4840561

[6] Murphy PG Myers DS Davies MJ Webster NR Jones JG The antioxidant potential of propofol (2,6-diisopropylphenol). Br J Anaesth 1992 68 613 8 10.1093/bja/68.6.613 1319189

[7] Laffey JG Boylan JF Cheng DC The systemic inflammatory response to cardiac surgery: implications for the anesthesiologist. Anesthesiology 2002 97 215 52 10.1097/00000542-200207000-00030 12131125

[8] Engels M Bilgic E Pinto A Vasquez E Wollschläger L Steinbrenner H A cardiopulmonary bypass with deep hypothermic circulatory arrest rat model for the investigation of the systemic inflammation response and induced organ damage. J Inflamm (Lond) 2014 11 26 10.1186/s12950-014-0026-3 25400510 PMC4231204

[9] Jiao Z Ma Y Wang Y Liu T Zhang Q Liu X Protective effect of adipose-derived mesenchymal stem cell secretome against hepatocyte apoptosis induced by liver ischemia-reperfusion with partial hepatectomy injury. Stem Cells Int 2021 2021 9969372 10.1155/2021/9969372 34457008 PMC8390152

[10] Dugbartey GJ Juriasingani S Zhang MY Sener AH _2_S donor molecules against cold ischemia-reperfusion injury in preclinical models of solid organ transplantation. Pharmacol Res 2021 172 105842 10.1016/j.phrs.2021.105842 34450311

[11] Toledo-Pereyra LH Simmons RL Najarian JS Protection of the ischemic liver by donor pretreatment before transplantation. Am J Surg 1975 129 513 7 10.1016/0002-9610(75)90308-6 1093421

[12] Lee EJ Silva SM Simões MeJ, Montero EF. Effect of N-acetylcysteine in liver ischemia-reperfusion injury after 30% hepatectomy in mice. Acta Cir Bras 2012 27 346 9 10.1590/S0102-86502012000400011 22534811

[13] Rancan EA Frota EI Freitas TMN Jordani MC Évora PRB Castro-E-Silva O. Evaluation of Indigo carmine on hepatic ischemia and reperfusion injury. Acta Cir Bras 2020 35 e202000901 10.1590/s0102-865020200090000001 32996998 PMC7518224

[14] Tanoglu A Artis T Donmez R Kargi A Sit M Aslan S Liver transplantation from living donors with Gilbert’s syndrome is a safe procedure for both donors and recipients. Clin Transplant 2015 29 965 43 10.1111/ctr.12615 26271485

[15] Busuttil RW Tanaka K The utility of marginal donors in liver transplantation. Liver Transpl 2003 9 651 63 10.1053/jlts.2003.50105 12827549

[16] Baskin-Bey ES Canbay A Bronk SF Werneburg N Guicciardi ME Nyberg SL Cathepsin B inactivation attenuates hepatocyte apoptosis and liver damage in steatotic livers after cold ischemia-warm reperfusion injury. Am J Physiol Gastrointest Liver Physiol 2005 288 G396 402 10.1152/ajpgi.00316.2004 15472011

[17] Lin Y Manning PT Jia J Gaut JP Xiao Z Capoccia BJ CD47 blockade reduces ischemia-reperfusion injury and improves outcomes in a rat kidney transplant model. Transplantation 2014 98 394 401 10.1097/TP.0000000000000252 24983310 PMC4887281

[18] Kim S Lee S Lee H Ju S Park S Kwon D A colon-targeted prodrug, 4-phenylbutyric acid-glutamic acid conjugate, ameliorates 2,4-dinitrobenzenesulfonic acid-induced colitis in rats. Pharmaceutics 2020 12 843 10.3390/pharmaceutics12090843 32899177 PMC7558321

[19] Liu S He L Yao K The antioxidative function of alpha-ketoglutarate and its applications. BioMed Res Int 2018 2018 3408467 10.1155/2018/3408467 29750149 PMC5884300

[20] Vilatoba M Eckstein C Bilbao G Smyth CA Jenkins S Thompson JA Sodium 4-phenylbutyrate protects against liver ischemia reperfusion injury by inhibition of endoplasmic reticulum-stress mediated apoptosis. Surgery 2005 138 342 51 10.1016/j.surg.2005.04.019 16153446

[21] Hlaváč V Holý P Souček P Pharmacogenomics to predict tumor therapy response: a focus on atp-binding cassette transporters and cytochromes P450. J Pers Med 2020 10 108 10.3390/jpm10030108 32872162 PMC7565825

[22] Huls M van den Heuvel JJ Dijkman HB Russel FG Masereeuw R ABC transporter expression profiling after ischemic reperfusion injury in mouse kidney. Kidney Int 2006 69 2186 93 10.1038/sj.ki.5000407 16612327

[23] Xiao Q Zhou Y Lauschke VM Ethnogeographic and inter-individual variability of human ABC transporters. Hum Genet 2020 139 623 46 10.1007/s00439-020-02150-6 32206879 PMC7170817

[24] Krishnamurthy P Schuetz JD Role of ABCG2/BCRP in biology and medicine. Annu Rev Pharmacol Toxicol 2006 46 381 410 10.1146/annurev.pharmtox.46.120604.141238 16402910

[25] Leslie EM Deeley RG Cole SP Multidrug resistance proteins: role of P-glycoprotein, MRP1, MRP2, and BCRP (ABCG2) in tissue defense. Toxicol Appl Pharmacol 2005 204 216 37 10.1016/j.taap.2004.10.012 15845415

[26] Bayramoglu G Bayramoglu A Engur S Senturk H Ozturk N Colak S The hepatoprotective effects of Hypericum perforatum L. on hepatic ischemia/reperfusion injury in rats. Cytotechnology 2014 66 443 8 10.1007/s10616-013-9595-x 23794084 PMC3973799

[27] Suakıtıcı S Güven BB Tanoglu A Özkan S A combination of levosimendan and N-Acetylcysteine shows significant favorable efficacy on experimental liver ischemia/reperfusion injury. Ulus Travma Acil Cerrahi Derg 2021 27 381 8 34213003 10.14744/tjtes.2020.81782

[28] Suzuki S Toledo-Pereyra LH Rodriguez FJ Cejalvo D Neutrophil infiltration as an important factor in liver ischemia and reperfusion injury. Modulating effects of FK506 and cyclosporine. Transplantation 1993 55 1265 72 10.1097/00007890-199306000-00011 7685932

[29] Cannistrà M Ruggiero M Zullo A Gallelli G Serafini S Maria M Hepatic ischemia reperfusion injury: A systematic review of literature and the role of current drugs and biomarkers. Int J Surg 2016 33 Suppl 1 57 70 10.1016/j.ijsu.2016.05.050 27255130

[30] Yang G Peng X Hu Y Lan D Wu Y Li T 4-phenylbutyrate benefits traumatic hemorrhagic shock in rats by attenuating oxidative stress, not by attenuating endoplasmic reticulum stress. Crit Care Med 2016 44 e477 91 10.1097/CCM.0000000000001469 26646458

[31] Dröse S Brandt U Wittig I Mitochondrial respiratory chain complexes as sources and targets of thiol-based redox-regulation. Biochim Biophys Acta 2014 1844 1344 54 10.1016/j.bbapap.2014.02.006 24561273

[32] Can M Guven B Bektas S Arikan I Oxidative stress and apoptosis in preeclampsia. Tissue Cell 2014 46 477 81 10.1016/j.tice.2014.08.004 25200618

[33] Ji C Kaplowitz N Hyperhomocysteinemia, endoplasmic reticulum stress, and alcoholic liver injury. World J Gastroenterol 2004 10 1699 708 10.3748/wjg.v10.i12.1699 15188490 PMC4572253

[34] Wang HC Wu HC Chen CF Fausto N Lei HY Su IJ Different types of ground glass hepatocytes in chronic hepatitis B virus infection contain specific pre-S mutants that may induce endoplasmic reticulum stress. Am J Pathol 2003 163 2441 9 10.1016/S0002-9440(10)63599-7 14633616 PMC1892360

[35] Liu L Wu H Zang J Yang G Zhu Y Wu Y 4-phenylbutyric acid reveals good beneficial effects on vital organ function via anti-endoplasmic reticulum stress in septic rats. Crit Care Med 2016 44 e689 701 10.1097/CCM.0000000000001662 26958745

[36] Liu J Ren F Cheng Q Bai L Shen X Gao F Endoplasmic reticulum stress modulates liver inflammatory immune response in the pathogenesis of liver ischemia and reperfusion injury. Transplantation 2012 94 211 7 10.1097/TP.0b013e318259d38e 22790388 PMC3414672

[37] Theodoulou FL Kerr ID ABC transporter research: going strong 40 years on. Biochem Soc Trans 2015 43 1033 40 10.1042/BST20150139 26517919 PMC4652935

[38] Karatas OF Guzel E Duz MB Ittmann M Ozen M The role of ATP-binding cassette transporter genes in the progression of prostate cancer. Prostate 2016 76 434 44 10.1002/pros.23137 26708806

[39] Liu DM Yang D Zhou CY Wu JS Zhang GL Wang P Aloe-emodin induces hepatotoxicity by the inhibition of multidrug resistance protein 2. Phytomedicine 2020 68 153148 10.1016/j.phymed.2019.153148 32028185

[40] Awad AS Elariny HA Sallam AS Colchicine attenuates renal ischemia-reperfusion-induced liver damage: implication of TLR4/NF-κB, TGF-β, and BAX and Bcl-2 gene expression. Can J Physiol Pharmacol 2022 100 12 8 10.1139/cjpp-2021-0007 34411492

[41] Trauner M Arrese M Soroka CJ Ananthanarayanan M Koeppel TA Schlosser SF The rat canalicular conjugate export pump (Mrp2) is down-regulated in intrahepatic and obstructive cholestasis. Gastroenterology 1997 113 255 64 10.1016/S0016-5085(97)70103-3 9207286

[42] Merino G van Herwaarden AE Wagenaar E Jonker JW Schinkel AH Sex-dependent expression and activity of the ATP-binding cassette transporter breast cancer resistance protein (BCRP/ABCG2) in liver. Mol Pharmacol 2005 67 1765 71 10.1124/mol.105.011080 15722455

[43] Hernández Lozano I Bauer M Wulkersdorfer B Traxl A Philippe C Weber M Measurement of hepatic ABCB1 and ABCG2 transport activity with (11C)tariquidar and PET in humans and mice. Mol Pharm 2020 17 316 26 10.1021/acs.molpharmaceut.9b01060 31790256

[44] Doyle MJ Maher TJ Li Q Garry MG Sorrentino BP Martin CM Abcg2-labeled cells contribute to different cell populations in the embryonic and adult heart. Stem Cells Dev 2016 25 277 84 10.1089/scd.2015.0272 26573225 PMC4742971

[45] Xiong H Callaghan D Jones A Bai J Rasquinha I Smith C ABCG2 is upregulated in Alzheimer’s brain with cerebral amyloid angiopathy and may act as a gatekeeper at the blood-brain barrier for Abeta(1-40) peptides. J Neurosci 2009 29 5463 75 10.1523/JNEUROSCI.5103-08.2009 19403814 PMC2745912

[46] Liu WH Liu HB Gao DK Ge GQ Zhang P Sun SR ABCG2 protects kidney side population cells from hypoxia/reoxygenation injury through activation of the MEK/ERK pathway. Cell Transplant 2013 22 1859 68 10.3727/096368912X657206 23032069

[47] Ogura J Kobayashi M Itagaki S Hirano T Iseki K Post-transcriptional regulation of breast cancer resistance protein after intestinal ischemia-reperfusion. Biol Pharm Bull 2008 31 1032 5 10.1248/bpb.31.1032 18451542

[48] Donner MG Topp SA Cebula P Krienen A Gehrmann T Sommerfeld A HbG200-mediated preinduction of heme oxygenase-1 improves bile flow and ameliorates pericentral downregulation of Bsep and Mrp2 following experimental liver ischemia and reperfusion. Biol Chem 2013 394 97 112 10.1515/hsz-2012-0153 23096566

[49] Thorling CA Liu X Burczynski FJ Fletcher LM Roberts MS Sanchez WY Intravital multiphoton microscopy can model uptake and excretion of fluorescein in hepatic ischemia-reperfusion injury. J Biomed Opt 2013 18 101306 10.1117/1.JBO.18.10.101306 23812606

[50] Ban D Kudo A Sui S Tanaka S Nakamura N Ito K Decreased Mrp2-dependent bile flow in the post-warm ischemic rat liver. J Surg Res 2009 153 310 6 10.1016/j.jss.2008.02.064 18662814

[51] Maher JM Cherrington NJ Slitt AL Klaassen CD Tissue distribution and induction of the rat multidrug resistance-associated proteins 5 and 6. Life Sci 2006 78 2219 25 10.1016/j.lfs.2005.09.016 16260000

[52] Borst P de Wolf C van de Wetering K Multidrug resistance-associated proteins 3, 4, and 5. Pflugers Arch 2007 453 661 73 10.1007/s00424-006-0054-9 16586096

